# Effectiveness of 1 and 2 per cent acetic acid solutions in the 2-week treatment of granular myringitis – ERRATUM

**DOI:** 10.1017/S002221512300155X

**Published:** 2023-11

**Authors:** S Prakairungthong, T Werawatganon, T Chantarawiwat, L Warnpeurch, S Atipas, K Thongyai, K Suvarnsit, S Limviriyakul

The publisher apologises that upon publication of this article the surname of author S Limviriyakul was incorrectly spelled as Limiviriyakul.

The online version of this article has been updated.
